# The implementation of European animal welfare standards in Turkish animal protection legislation and case law

**DOI:** 10.3389/fvets.2026.1852760

**Published:** 2026-08-31

**Authors:** Çağdan Uyar

**Affiliations:** Department of Forestry, Vocational School of Forestry, Istanbul University-Cerrahpaşa, Istanbul, Türkiye

**Keywords:** animal rights, animal welfare, biodiversity protection, court decisions, environmental law, judicial gap, law no. 5199

## Abstract

**Introduction:**

In this study, by examining the compatibility of Turkish legislation with the European convention on animal welfare through quantitative analysis and case law, the purpose is to fill a significant gap in the literature.

**Methods:**

The study employs a mixed-method approach encompassing quantitative legislative evaluation, case law review, and a strategic SWOT analysis. The compatibility of national laws with the Convention was quantitatively measured. Furthermore, a comprehensive analysis of 250 Supreme Court decisions was systematically conducted to assess the practical implementation and reflection of these international norms within the judicial system.

**Results:**

The findings reveal that Turkish legislation is generally 77% compliant with the Convention, indicating a level of compliance that is sufficient but could be improved. While full compliance is observed in areas such as the keeping of animals and surgical operations on animals, significant shortcomings persist in areas such as animal sanctuaries, trading, and commercial breeding. The case law analysis highlights a critical gap between international norms and judicial practice; only 3.6% of the examined decisions directly reference the Convention. Furthermore, only 33% of the cases that did reference the Convention resulted in outcomes in favor of animals; the majority of adverse outcomes arose from conflicts related to keeping animals in communal living areas.

**Discussion:**

By utilizing a strategic SWOT analysis, the study offers practical recommendations to address existing challenges. While presenting a clear roadmap to align national judicial practices with international animal welfare standards, the research also identifies the intrinsic relationship between judicial decisions, practical shortcomings, and the structural limitations within the Convention itself.

## 1 Introduction

The relationship between humans and animals has developed through a long his-torical and evolutionary process extending back thousands of years, during which an-imals have been regarded as companions, sources of food, labor, transportation, and scientific research subjects (1). As societies became increasingly industrialized and the use of animals expanded, ethical concerns regarding the treatment of animals gradually entered social, political, and legal discourse. This transformation contributed to the emergence of legal frameworks aimed at preventing cruelty and establishing re-sponsibilities toward animals (1, 2).

The origins of modern animal welfare legislation are generally associated with 19-century Britain, where Parliament became one of the first legislative au-thorities to recognize that animals could be legitimate subjects of legal protection. The Cruel Treatment of Cattle Act 1822 (Martin's Act) represented a significant milestone by introducing criminal sanctions against the cruel treatment of certain domestic animals. This Act marked an important transition from a purely property-based understanding of animals toward the recognition that unnecessary cruelty inflicted upon animals required legal intervention. The subsequent Cruelty to Animals Act 1835 substantially expanded this protection by extending legal safeguards to additional categories of animals, pro-hibiting practices such as animal fighting, and strengthening measures against unnec-essary suffering. Although 19-century animal protection laws were also influ-enced by concerns regarding public morality and social order, their development cannot be explained solely through human-centered interests. Rather, these legislative devel-opments represented an early recognition that animals themselves were worthy of protection from unnecessary pain and suffering and formed the foundation of modern British animal welfare law (2, 38).

The historical trajectory of animal protection law in the United States developed differently from that of Britain and continental Europe. While British and European approaches gradually evolved toward recognizing animal suffering and sentience as legitimate concerns of legal protection, American animal protection law developed primarily through state-level legislation and was strongly influenced by utilitarian and anthropocentric considerations. In the 19th century, legal protection of animals in the United States was often associated with preventing cruelty considered harmful to public morality, human interests, or social order, while animals continued to be pre-dominantly regarded as property under legal systems. Therefore, although both Euro-pean and American legal traditions addressed animal cruelty, their conceptual founda-tions and historical development differed significantly (2–4).

The 20th century witnessed a transformation in scientific and ethical approaches toward animals. The emergence of animal welfare science demonstrated that animals are sentient beings capable of experiencing pain, suffering, and positive affective states. The publication of the Brambell Report in 1965 represented an important milestone in this development by emphasizing that animal welfare should not be limited to the prevention of physical suffering but should also consider animals' behavioral needs and opportu-nities to express natural behaviors (9). Consequently, contemporary animal welfare legislation increasingly seeks not only to prevent cruelty but also to es-tablish positive welfare standards based on scientific evidence regarding animals' bio-logical, behavioral, and psychological needs (5, 7, 8).

It is important to distinguish animal welfare from animal rights. Animal welfare permits the use of animals by humans, provided that their physical and psychological wellbeing is protected and unnecessary suffering is avoided. In contrast, animal rights theory maintains that animals possess inherent rights that should not be violated, irre-spective of the benefits derived from their use (37).

Considering that animals' life-history strategies are shaped by demographic pa-rameters such as growth rate, reproductive timing, mortality patterns, and lifespan (6), the extent to which scientific knowledge regarding animal welfare is incorporated into legal systems remains an important subject of legal and ethical debate. Modern welfare assessment has therefore expanded beyond the absence of suffering and increasingly considers animals' affective experiences, behavioral opportunities, and overall quality of life. Nevertheless, many legal systems continue to rely on broad concepts such as pain, distress, and unnecessary suffering, creating continuing discus-sions regarding the adequacy of existing legal protection mechanisms (7, 8).

The gradual transformation of animals' legal status from property toward recogni-tion as sentient beings has encouraged significant reforms in national legal systems. Several jurisdictions, including France (2015), New Zealand (2015), and Spain (2021), have introduced legislative reforms recognizing animals as beings with interests distinct from ordinary property. Türkiye has also experienced a significant legal transformation through Law No. 7332, which entered into force in 2021 and amended the Animal Pro-tection Law No. 5199. Through this amendment, animals were removed from the tradi-tional classification of property under Turkish law and were legally recognized as living beings requiring protection (17).

Within this framework of global developments in animal protection law, this study evaluates the compliance of Türkiye's current legislation and judicial practices with the principles established under the European Convention for the Protection of Pet Animals (ECPPA). The study aims to examine whether Turkish legislation provides an adequate level of protection consistent with the obligations and principles arising from this in-ternational legal instrument.

### 1.1 The history and significance of the convention

The international development of animal welfare legislation in Europe is closely associated with the activities of the Council of Europe, an international organization es-tablished in 1949 to promote human rights, democracy, and the rule of law. Unlike the European Union, the Council of Europe does not possess supranational legislative aut-hority or a general coercive enforcement mechanism. Instead, it develops international conventions that become legally binding for member States that voluntarily sign and ratify these instruments according to their constitutional procedures. Consequently, the implementation of Council of Europe conventions depends largely on their incorporation and application within national legal systems (36).

The Council of Europe played a pioneering role in establishing international legal frameworks specifically addressing animal protection. Its treaty-based approach began with the European Convention for the Protection of Animals during International Transport (ETS No. 65), opened for signature in 1968. This was followed by a series of specialized conventions addressing different areas of animal protection, including “the European Convention for the Protection of Animals Kept for Farming Purposes (ETS No. 87, 1976), the European Convention for the Protection of Animals for Slaughter (ETS No. 102, 1979), the European Convention for the Protection of Vertebrate Animals Used for Experimental and Other Scientific Purposes (ETS No. 123, 1986), and finally the ECPPA (ETS No. 125, 1987)” (12, 13).

Before the establishment of binding international animal welfare treaties, several ethical initiatives contributed to the development of international awareness regarding animal protection. Among these initiatives, the Universal Declaration of Animal Rights, proclaimed in Paris in 1978 by the International League for Animal Rights and later presented to UNESCO, became an influential normative statement regarding the moral consideration of animals. However, the Declaration does not constitute a legally binding international instrument and does not create enforceable obligations under international law. Therefore, its significance lies primarily in its normative and educational influence rather than in establishing international legal duties (39).

The ECPPA (ETS No. 125) represents a significant development because it is the first international treaty specifically dedicated to companion animals. The Convention was opened for signature in Strasbourg on 13 November 1987 and entered into force on 1 May 1992 after achieving the required number of ratifications. It establishes minimum stan-dards concerning the keeping, breeding, trade, and responsible ownership of companion animals. The Convention reflects a shift away from viewing companion animals solely as objects of ownership and toward recognizing them as living beings with biological and behavioral requirements that require legal protection (14).

The Convention consists of provisions concerning general principles, the keeping of companion animals, supplementary measures for stray animals, information and edu-cation, and consultation mechanisms. It is open to accession by non-member States of the Council of Europe, demonstrating its broader international relevance. Türkiye signed the Convention on 18 November 1999 and ratified it through Law No. 4934 on the Approval of the Ratification of the ECPPA, published in the Official Gazette on 22 July 2003. Ac-cording to Article 90 of the Constitution of the Republic of Türkiye, duly ratified inter-national treaties acquire the force of law within the Turkish domestic legal order. The-refore, the ECPPAconstitutes part of Türkiye's domestic legal framework and provides an important reference point for evaluating national legislation concerning companion animal protection (11, 35).

### 1.2 Legal provisions regarding animal rights in Turkish legislation

In Turkish law, legal regulations regarding animal rights must first be examined in terms of Constitutional provisions. There is no direct provision regarding animal rights in Turkish constitutions. Article 56 of the 1982 Constitution mandates the improvement of the environment and the protection of environmental health. Apart from this, there is Article 45 regarding the development of animal husbandry, and Article 169 contains a regulation regarding the removal of forests useful for animal husbandry from forest status. Therefore, it can be stated that there is a deficiency in the Turkish constitution in this regard. For many years, a relevant law regarding the protection of animal rights did not enter into force. Only certain regulations were made regarding the protection of wild animals, but a substantial regulation regarding the protection of pets was not made until 2004. The law on the protection of animals, which entered into force in 2004, contains substantial regulations regarding the protection of pets. The most important criticism regarding this law has been the issue of penal sanctions regarding the killing of animals. Because the sanction regarding the killing of animals was regulated in the Turkish Penal Code (TPC), and animals were considered as property. As a result of public pressure on this issue, an amendment was made to Law No. 5199 in 2021, animals were removed from the status of property, and the sanction was transformed into a sanction regarding harm to a living being (15–18).

In accordance with Article 90 of the Constitution, Turkish law also accepts international conventions as a law. The ECPPA, to which Türkiye is a party, entered domestic law as a law from the date it was acceded to and is as binding as a law. According to Article 90 of the Constitution, international conventions carry the force of law. Paragraph 5 of Article 90 of the 1982 Constitution stipulates that international agreements put into effect duly carry the force of law, and that an appeal cannot be made to the Constitutional Court with the claim of unconstitutionality concerning them. In the continuation of the article, it is stated that in conflicts that may arise due to international agreements regarding fundamental rights and freedoms put into effect duly and domestic laws containing different provisions on the same issue, the provisions of the international agreement shall prevail. As can be understood, in Turkish law, international conventions to which Türkiye is a party are more effective legal regulations than domestic laws (16).

#### 1.2.1 The legislative process of Law No. 5199

The legal regulations starting with the “Animal Improvement Law” No. 904 accepted in 1926, primarily aimed at the development of animal husbandry, combating epidemic diseases, and determining the duties and authorities of veterinarians. Legal regulations regarding the maltreatment of animals and the damages caused by animals, on the other hand, were evaluated within the scope of the TPC No. 5237, which was adopted in 2004. According to the TPC, crimes committed against animals are regulated under the headings of qualified theft (Article 142), damage to property (Article 151), Intentional Pollution of the Environment (Article 181), and Negligent Pollution of the Environment (Article 182). Furthermore, the seizure and leasing of animals (property law) are also addressed in the Turkish Civil Code No. 4721 and the Turkish Code of Obligations No. 6098. Within the scope of the Misdemeanors Law No. 5236, it is seen that animal slaughtering and its waste are evaluated in terms of the crime of polluting the environment. The protection of endangered species is guaranteed in the Environment Law No. 2872. While the works regarding stray animals fell within the duties and responsibilities of municipalities until 1991, with the establishment of the Ministry of Environment on this date, the responsibility was shared between these two institutions. Türkiye became a party to the ECPPA in 2003, and approximately 1 year after this date, the Animal Protection Law No. 5199 was adopted and published in the Official Gazette dated July 1, 2004, and numbered 25509 (10, 11, 15, 16, 19). In this initial version of the law, the obligation to receive training was not imposed on people who would adopt and sell animals, and in many respects, crimes committed against animals remained unpunished. With the regulations made in Law No. 5199 in 2021, along with the increase in existing administrative fines and the added criminal penalties, it has become more deterrent in the protection of animals. The removal of animals from the scope of property and the expansion of the scope of criminal penalties have ensured an approach to the concepts of “welfare” and “rights” in the Convention. In addition to this, with the amendments made in 2021, the definitions of “companion animal,” “animal shelter,” and “rehabilitation” were added to the law, and the policy area of interest was partially expanded. A substantial amendment was made with Law No. 7332 in 2022 and with Law No. 7527 in 2024. In particular, while the killing of animals was previously subject to a fine, it has now been converted into a prison sentence (Article 28/A) (17, 20).

The Animal Protection Law No. 5199 dated 24.6.2004, which is the fundamental law regarding companion animals; this law aims to ensure the comfortable lives of animals and their good and appropriate treatment, to ensure their best protection against suffering pain, distress, and torment, and to prevent all kinds of their victimization. Although the law primarily aims to take companion animals under protection, along with this, it also carries the aim of ensuring the protection of all animals and their care under appropriate conditions (15, 19).

Another legislation that subjects companion animals is the Law on Veterinary Services, Plant Health, Food and Feed No. 5996 dated 11.06.2010. The aim of this law is to protect and ensure food and feed safety, public health, plant and animal health, as well as animal breeding and welfare, taking into consideration consumer interests and the protection of the environment. In Article 2 of the Law, birds are also expressed within the definition of companion animals. In Article 13 of the Law regarding birds, it is stated that in workplaces selling ornamental birds permitted by the Ministry, only veterinary medicinal products specific to these animals, excluding veterinary biological products, can be sold.

When the secondary legislation regarding companion animals is examined, some of the following regulations stand out:

- Implementing Regulation on the Protection of Animals published in the Official Gazette dated 13.12.2024 and numbered 32751; this regulation covers the arrangements to be made and the measures to be taken in line with the objectives specified in Article 1, and it does not cover the implementations related to the acts within the scope of the crimes stipulated in Article 28/A of Law No. 5199.
- Regulation on the Breeding, Sale, Housing, and Training Places of Pet Animals published in the Official Gazette dated 08.10.2011 and numbered 28078; the aim of this regulation is to regulate the procedures and principles regarding the necessary technical and health conditions and the opening, working, and inspection of the places where companion animals are bred, bought and sold, housed, and trained.
- Road Transport Regulation published in the Official Gazette dated 08.01.2018 and numbered 30295; this regulation regulates the procedures regarding the transportation of animals.
- With the Regulation on the Identification and Registration of Cats, Dogs, and Ferrets published in the Official Gazette dated 26.02.2018 and numbered 30344, regulations have been made to ensure the tracking of animal movements and the control of their diseases at a more effective level.
- Regulation on the Implementation of the Convention on International Trade in Endangered Species of Wild Fauna and Flora (CITES) published in the Official Gazette dated 27.12.2001 and numbered 24623; this regulation contains provisions regarding the control of international trade in order to ensure the sustainable use of animal and plant species within the scope of the CITES convention.
- The aim of the Animal Health and Policing Regulation published in the Official Gazette dated 15.03.1989 and numbered 20109 is to protect against diseases that can be transmitted from animals and animal substances to humans and animals, and to determine the principles and procedures of combating infectious animal diseases.

Furthermore, there is a brief article regarding stray animals in the 11th Development Plan of the Republic of Türkiye, covering the years 2019–2023; “Mechanisms will be established to ensure the effectiveness of regulations and measures to be taken for stray animals, and the rehabilitation projects of local governments for stray animals will be supported” (21). As stated in the 12th Development Plan of the Republic of Türkiye, covering the years 2024–2028; food security and safety can only be achieved in an environment where animal health is guaranteed and sustainable animal husbandry is established. Evaluating animal health, animal welfare, and human health holistically together is increasingly turning into an unavoidable necessity in the world. Municipalities with a population of less than 25,000, which do not have the obligation to establish an animal shelter, have also been obliged to take the stray animals in their area of responsibility to the nearest municipality to have them rehabilitated (22).

## 2 Materials and methods

**Figure 1:**
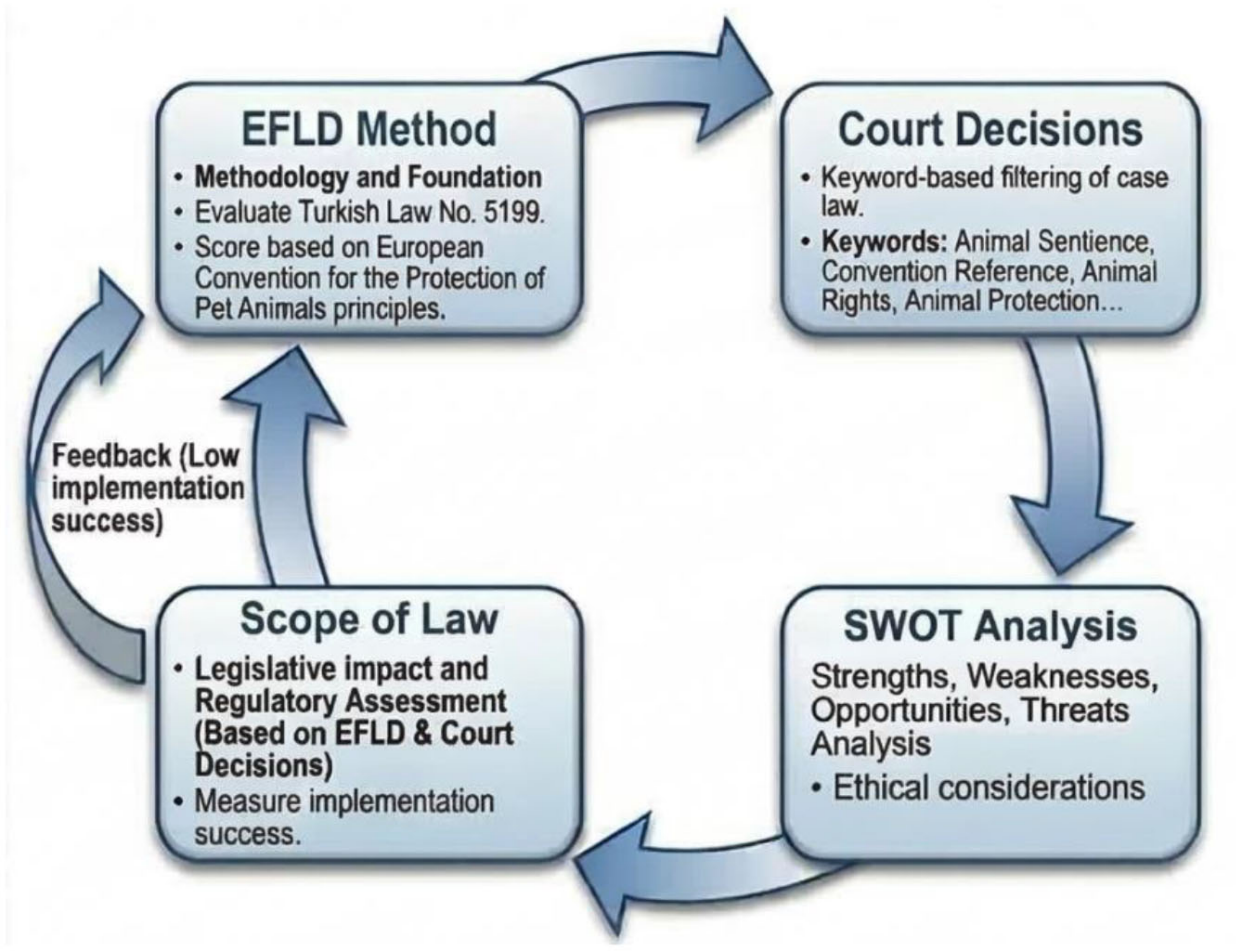
Methodological cycle for animal rights analysis.

As a research method, a cyclical research method (Figure 1) acting as a validator of the measurements, which follow each other and then return to the beginning, was selected (Generated with the assistance of Gemini 3.1 Pro. The final content and scientific accuracy were verified). According to the validating methodological cycle; if the obtained EFLD score is low, the power of the law is considered low, and the next stage is not proceeded to. Recommendations are given regarding the deficiencies that need to be eliminated in order to restart the evaluation. However, if the implementation power of the law is found to be high according to the EFLD scoring, the cycle continues, and a contribution is made to the improvement of the current situation by making recommendations only for the improvement of the lacking aspects. Comprehensive methodology cycle, including Law No. 5199 evaluation, EFLD, keyword basis case law analysis, SWOT, and feedback loop for low implementation success. It is as follows:

Initially, Web of Science articles related to the subject were examined. In the publications made to date regarding the effectiveness of the Convention in Türkiye and in Turkish publications, it has been observed that this study has not been conducted before. In order to determine the compliance level of the legislation in question with scientific and objective data in the research, the EFLD (Environmental and Forest Law Department) Method, which offers the possibility of systematic scoring in legal analyses, was applied. The research was not strictly limited to normative regulations; by examining the judicial decisions selected through keywords related to the subject, the effectiveness of the existing legal texts in the implementation phase and their equivalence in domestic law were also meticulously scrutinized. The data obtained through this method were supported by a simultaneously conducted SWOT analysis, and the accuracy of the results was ensured. In this direction; international good practice examples, comprehensive literature review, and legal gap analysis were synthesized, and concrete solution proposals were developed in the light of the findings obtained.

According to this cyclical methodology, the control mechanism has a dynamic structure. Specifically, if the measurement of international convention and law to be assessed using the EFLD is above the threshold value of 50%, the process moves on to the court decisions stage. The number of citations in these decisions and the ratio of positive to negative outcomes are determined, and then a SWOT analysis is conducted based on each country's current approaches and institutional and social structures. From this point onward, the success of implementing legal regulations and court decisions is evaluated. Each cycle begins again with the EFLD phase. This cycle can be reapplied for analysis with every new development (such as new legislative amendments or the re-evaluation of court decisions) and will be decisive in showing the progress made compared to the past. This method is designed to ensure that future legislative and judicial practices are consistently more successful. The step-by-step explanation of the applied methodological approach is as follows:

To analyse legal regulations and policy texts, the EFLD methodology, which is widely accepted in the literature, was used. In accordance with this method, its content was analyzed by applying the EFLD scoring method to each article of Law No. 5199 in terms of its compliance with the determined 12 principles of the Convention. In order to examine whether a specific criterion contributing to the protection of companion animals is at a satisfactory level, the principles were analyzed for each article of the law and scored according to the level of protection. Following the scoring, the content of the Law for each principle was explained and shown in separate groups. Furthermore, by identifying and evaluating the legal deficiencies in this regard, it was determined which principles do not have a sufficient impact on companion animals (23–25). Thus, the reflections of the theoretical legal framework on practical management decisions and judicial review were evaluated with a holistic perspective. A five-point scoring system, namely the EFLD method, was used for scoring (Table 1). This criterion has been previously used by environmental and forest lawyers (26–31).

**Table 1:** EFLD method scoreboard.

Point	Description
0	No provision
1	Very general expressions; insufficient
2	Similar expressions exist, but they are insufficient
3	No exact equivalent, but sufficient
4	High similarity

In this method of social analysis, the provisions of the relevant legislation pertaining to the research topic are examined in four stages. Since the explanation and interpretation of each law's articles differ from one another, their interpretation varies depending on the legal experts' mastery of the legislation. In this study as well, the law was meticulously examined in its entirety, and the criteria corresponding to each point were assessed for their presence in the relevant legal provision, and the law was scored accordingly. International conventions analyzed using this method have previously been published in peer-reviewed journals. In these articles, a threshold of 50% compatibility was adopted as the standard for this method. Legal documents showing compatibility above 50% based on the scoring criteria are evaluated as sufficient/valid. The scoring table of the method is as follows in Table 1.

According to Law No. 5199, the topics examined and scored under the Convention Principles are as follows.

Animal welfare, Keeping of animals, Breeding of animals, Conditions for acquiring a companion animal, Training of companion animals, Trading and commercial breeding, Animal sanctuaries, Subjecting animals to advertising, entertainment, exhibitions and competitions, Surgical operations on animals, Killing of animals, Reduction of animal numbers, Information and education programs about animals.

- The analysis of judicial decisions in the study was conducted over 250 Turkish supreme court (Yargitay) decisions from the last 10 years (between the years 2015–2025). The decisions of the Turkish Court of Cassation were examined in terms of the subjects of animal rights and convention principles. In the research conducted on a special jurisprudence search program (Kazanci jurisprudence search engine/Kazanci Ictihat Bilgi Bankasi), which provides researchers and jurists with access to precedent judicial decisions, the keywords “*the European Convention for the Protection of Pet Animals, companion animal rights, companion animal welfare, companion animal trade, companion animal shelters, killing of companion animals, protection of companion animals, companion animal theft*” were searched. Among these judicial decisions, the court decisions referring to the international convention were examined (27, 28, 32, 33). From the court decisions examined until the final sample was obtained, 216 concerned animal theft and 25 concerned the killing of animals. Only nine court decisions that directly cited the ECPPA were accessed. An evaluation was made regarding the positive or negative impact of the referring court decisions on animal welfare.
- After examining the judicial functionality of Türkiye's efforts to protect animal rights, a SWOT analysis was conducted in terms of Turkish legislation and practice by adhering to the provisions of the Convention. Prior to the analysis, the outputs of academic meetings attended, official institution data, interviews with subject expert scientists, the legal content analysis conducted, mainstream media news, social responsibility project reports, etc. were evaluated together. In the resulting table, action-oriented strategies were produced by bringing together internal factors (Strengths/Weaknesses) and external factors (Opportunities/Threats). This table was compiled using statements from the Ministry of National Education's official website and social media accounts, news reports published in the Turkish press regarding NGOs, Türkiye's accession to the international convention in 2003, and, regarding experts, both the sources referenced in the article and expert opinions obtained through face-to-face interviews with three faculty members from the Department of Environmental and Forest Law, who are both legal scholars and foresters, were utilized. Legal analysis involved an examination of Law No. 5199 and the relevant provisions of the international convention. The work conducted by the Animal Rights Commission of the Union of Turkish Bar Associations, along with the outcomes and proceedings of its meetings, were reviewed (e.g., hayvanhaklari.barobirlik.org.tr).
- Together with the findings obtained as a result of the content analysis in the law and the SWOT analysis examined in line with the implementation of the convention, a joint evaluation of the general literature and the main findings was made, and policy recommendations were given in the direction of the protection of animals.

## 3 Results

The results of the study were examined under the headings of EFLD analysis, analysis of judicial decisions, and SWOT analysis.

### 3.1 EFLD analysis

The compliance of the 12 articles constituting the main headings within the content of the Convention with Law No. 5199 and their effectiveness in protecting animals have been measured in the Table 2.

**Table 2:** Compatibility between the articles of the law and the international convention.

Convention principles	Relevant articles of Turkish law (5199)	EFLD point
Article 3: animal welfare	Article (Art.) 4: the fundamental principles regarding the protection and comfortable lives of animals have been determined. Art. 4/g: in ensuring the protection and comfortable lives of animals; the hygiene, health, and safety of humans and other animals must also be taken into consideration. Art. 4/k: animal owners must perform sterilization and digital identification. Art. 5: the adoption, care, and protection of animals (a companion animal kept without a commercial purpose cannot be seized due to the debt of its owner). Art. 10: animal trade (transportation) has been regulated. Art. 13/3: a regulation has been made regarding the euthanasia of animals (... except in emergencies, animals cannot be killed during the periods of giving birth, pregnancy, and nursing). Art. 14: prohibitions regarding animals have been determined. Art. 20: the subject of educational publications has been determined. Art. 22: it is about the subject of zoos and natural life parks (business owners and municipalities must provide optimal living conditions). Art. 28/A: it regulates criminal penalties as fines and imprisonment sentences.	4
Article 4:keeping of animals	Art. 3: definitions (companion animal). Art. 4: the fundamental principles regarding the protection and comfortable lives of animals have been determined. Art. 7: surgical interventions (interventions on animals are performed only by veterinarians). Art. 10: while being sold; it is mandatory that the health of the animals is good, and the place where they are housed is clean and suitable for health conditions. During the care, feeding, transportation, and slaughtering of farm animals; health conditions must be complied with in the breeding and trade of companion animals. Art. 11: animals cannot be trained in a way that exceeds their capacity and strength, and making them fight with other living beings is prohibited. Art. 14: prohibitions (the care of animals cannot be neglected, companion animals cannot be abandoned, stray animals cannot be abandoned anywhere other than an animal shelter). Art. 24: taking under protection (individuals who neglect animal care, inflict pain, suffering, and harm are officially prohibited from acquiring animals, and those animals that can be adopted are taken to an animal sanctuary). Art. 28: administrative fines (an administrative fine per animal is applied to individuals and businesses who do not take all precautions regarding animal adoption and care, and who cause disturbance and harm). Art. 28/A: it regulates criminal penalties as fines and imprisonment sentences.	4
Article 5: breeding of animals	Art. 10: trade of animals (sale from breeding places). Art. 10/4: those who breed and trade companion animals are obliged to take the necessary precautions regarding anatomical, physiological, and behavioral characteristics in order not to endanger the health of the animals. Art. 13/3: except for legal exceptions, medical and scientific justifications, and emergencies posing unavoidable threats to human and environmental health that are not for food purposes, animals cannot be killed during the periods of giving birth, pregnancy, and nursing. Art. 14: prohibitions regarding animals have been determined. Art. 14/f: to slaughter or kill animals other than game animals permitted to be hunted and bred as slaughter animals in private breeding farms, and wild animals subject to trade, for the purpose of meat need and to put them on the market. Art. 14/h: to obtain fetuses, caviar, and similar products from the animal's womb without legal or medically justified permission. Additional Article 2: responsibility of local governments (animals cannot be bred without the permission of the ministry, their ethologies are observed).	4
Article 6: conditions for acquiring a companion animal	Art. 4: principles (adopting animals solely with humanitarian and conscientious responsibility, digital identification, ensuring the hygiene, health, and safety of animals). Art. 5: the adoption, care, and protection of animals (undertaking the care of the animal in accordance with its ethology by taking precautions regarding human and environmental health). Art. 14: prohibitions have been regulated (the breeding, adoption, gifting, feeding, trade, and advertisement of animals determined to pose a danger by the Ministry are prohibited). Art. 14/d: it is prohibited to sell companion animals to those under the age of 16.	4
Article 7: training of companion animals	Art. 3: definitions (companion animal). Art. 11: training (Animals cannot be trained in a way that exceeds their natural capacity or strength, or with methods that will cause them injury, unnecessary suffering, or encourage bad habits). Art. 28: regulates administrative fines.	4
Article 8: trading and commercial breeding	Art. 5: the adoption, care, and protection of animals (individuals selling companion animals are obliged to obtain a certificate by participating in the care and protection training programs organized by local governments). Art. 10: trade of animals (While being sold; it is mandatory that the health of the animals is good, and the place where they are housed is clean and suitable for health conditions. Matters related to the use of animals for commercial purposes in filming and advertising are subject to the permission of the Ministry). Art. 14: prohibitions have been determined (the entry into the country and sale of animals... determined to pose a danger by the Ministry are prohibited).	2
Article 8: animal sanctuaries	Art. 4: principles (their care in animal sanctuaries and, if possible, in animal hospitals by local governments and voluntary organizations until they are adopted). Art. 6: protection of stray and debilitated animals (animals taken to animal sanctuaries are registered in the Ministry database and housed there until they are adopted. Furthermore, buildings and facilities suitable for the purpose may be allocated by official institutions, with the permission of the Ministry, to natural and legal persons who will establish animal shelters by adopting animals solely for humanitarian and conscientious purposes without profit and benefit, provided that the ownership remains with the administrations). Art. 19: financial support (financial support is provided in amounts deemed appropriate by the Ministry to establish animal shelters and hospitals aimed at protecting human, animal, and environmental health, to provide medicine, equipment, etc., and to carry out adoption activities).	1
Article 9: subjecting animals to advertising, entertainment, exhibitions and competitions	Art. 10: trade of animals (Matters related to the use of animals for commercial purposes in filming and advertising are subject to the permission of the Ministry. An animal cannot be used for filming, shows, advertising, and similar works in a way that will cause it pain, suffering, or harm). Art. 11: training (It is prohibited to make animals fight with another living animal. Traditional shows for folkloric purposes that do not contain violence can be organized by obtaining official permission). Art. 14: prohibitions (Exhibiting, advertising... animals determined to pose a danger by the Ministry is prohibited). Provisional Art. 3: penalties have been regulated.	2
Article 10: surgical operations on animals	Art. 4: principles (sterilization of cats and dogs by their owners in communal living areas). Art. 7: medical and surgical interventions on animals are performed only by veterinarians. In order to prevent uncontrolled breeding, sterilization interventions are performed on animals without causing them pain.	4
Article 11 and 13: killing of animals	Art. 6: the killing of stray or debilitated animals is prohibited, except for legal exceptions. Art. 12: slaughter of animals (the slaughter of animals or sacrificial animals for religious purposes is carried out without frightening them, in a way that causes the least pain, hygienically and in accordance with the procedure, by qualified persons instantly). Art. 13: euthanasia of Animals (Except for legal exceptions, medical and scientific justifications, and emergencies posing unavoidable threats to human and environmental health that are not for food purposes, animals cannot be killed during the periods of giving birth, pregnancy, and nursing). Art. 14: regulations have been made regarding prohibitions. Art. 28/A: it regulates criminal penalties as fines and imprisonment sentences. Additional Article 1: a penalty has been regulated regarding the fact that the allowance for the collection of stray animals is under the responsibility of local governments, and regulations have also been made in Provisional Art. 4 regarding this issue.	4
Article 12: reduction of animal numbers	Art. 13: euthanasia of Animals (Except for legal exceptions, medical and scientific justifications, and emergencies posing unavoidable threats to human and environmental health that are not for food purposes, animals cannot be killed during the periods of giving birth, pregnancy, and nursing). Additional Article 2: the responsibility of local governments has been explained.	2
Article 14: information and education programs about animals	Art. 4: principles (It determines the obligations of local governments and voluntary organizations). Art. 5: the adoption, care, and protection of animals (individuals selling companion animals are obliged to obtain a certificate by participating in the care and protection training programs organized by local governments. The procedures and principles of the training to be provided regarding the care and protection of companion animals are determined by a regulation issued by the Ministry). Art. 14: it regulates that animals must not be sold by untrained individuals and must not be sold to those under the age of 16. Art. 16: it has been determined that the provincial animal protection board must organize educational activities regarding the love, protection, and keeping alive of animals. Art. 20: educational publications (for the purpose of animal protection and welfare, organizing programs for formal and non-formal education, giving place to this issue in the press and media; it is mandatory to make educational broadcasts on the programs of the Turkish Radio and Television Corporation and private television channels for the periods determined annually at times with the highest audience rating).	2

When the scoring results of the EFLD method, which was taken as a basis as a result of the examination of the law articles according to the Convention principles, are evaluated, the compliance of Turkish Legislation with the convention has emerged as 77%. This rate is evaluated as a sufficient rate in terms of the compliance of Turkish legislation with the international convention (23, 29, 30). It has been observed that Turkish legislation shows full compliance with the convention's principles titled Animal welfare, Keeping of animals, Breeding of animals, Conditions for acquiring a companion animal (age limit), Training of companion animals, Surgical operations on animals, Killing of animals, and that Turkish legislation is sufficient in this regard. It is possible to state that it does not show full compliance in terms of the principles titled Trading and commercial breeding, Animal sanctuaries, Subjecting animals to advertising, entertainment, exhibitions and competitions, Reduction of animal numbers, Information and education programs about animals, and that there are inadequacies and legislative deficiencies in this regard. The determinations regarding the inadequacies in question are as follows.

**The principle of animal sanctuaries:** according to the Convention, the establishment and notification of private sanctuaries are requested. This situation has not been explicitly regulated in Law No. 5199, and a publicly-dominated system has been determined regarding the establishment of sanctuaries. Due to public resources also suffering from budget shortages in this regard, sanctuaries have deficiencies in terms of number, capacity, and benefit.

**The principle of Trading and commercial breeding:** it is stated in the Convention that experienced and trained individuals can breed animals; and that they must report the animal species, property, and equipment to the competent authorities. There is no explicit provision or regulation in Law No. 5199 on this matter.

**The principle of subjecting animals to advertising, entertainment, exhibitions and competitions:** there is no concession in the Convention regarding this matter. Although the use of animals in matters such as exhibitions and competitions is prohibited in Law No. 5199, subjecting animals to “traditional shows for folkloric purposes that do not contain violence” in a way that can breach this principle and compromise on this principle is a significant concession. According to the provisions of this article in Türkiye, bull and camel wrestling are held, and it is observed that animals cause serious harm to each other in these competitions.

**The principle of reduction of animal numbers:** there are provisions regarding the killing of animals in the reduction of their numbers in the Convention. In Article 13 of Law No. 5199 as well, the method of reducing numbers by killing animals when they pose a danger to environmental and human health is resorted to. However, not all of the reduction conditions in the convention find equivalence in Law No. 5199.

**The principle of information and education programs about animals:** it is explained in the Convention on which subjects the education should be conducted. In this article, it is regulated that animal trainers must be competent and knowledgeable individuals, that they cannot be gifted to those under the age of 16 without parental responsibility, that animals cannot be given as prizes, that they cannot be bred unplanned, that wild animals cannot be kept as companion animals, and that these irresponsibilities can lead to the abandonment of animals. However, in Law No. 5199, under the title of educational publications, the implementation of education programs has been regulated, but it has not been specified which subjects the education will highlight.

### 3.2 Analysis of judicial decisions

**Figure 2:**
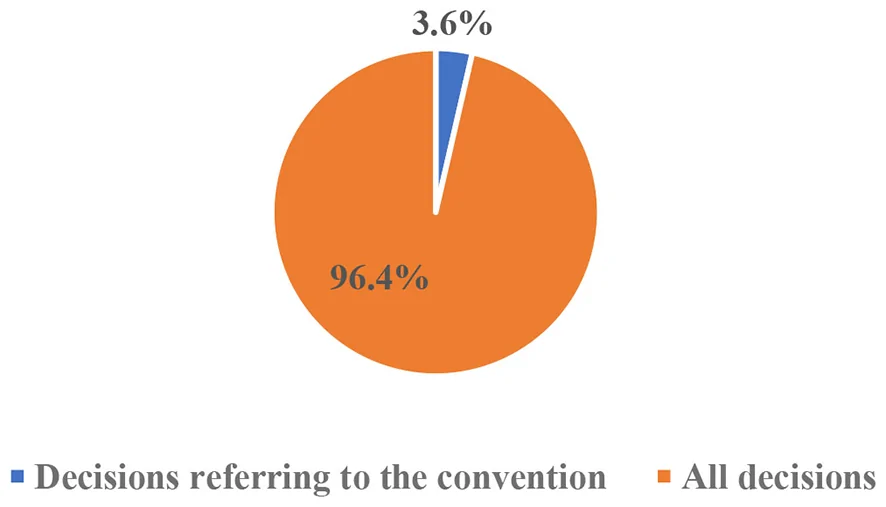
The percentage of Supreme Court decisions that cite the convention.

As stated in the methodology section, the analysis of judicial decisions in the study was conducted over Turkish supreme court decisions. The decisions of the Turkish Court of Cassation were examined in terms of the subjects of animal rights and convention principles. In the research conducted on a special jurisprudence program that provides researchers and jurists with access to precedent judicial decisions, the keywords “*the European Convention for the Protection of Pet Animals, companion animal rights, companion animal welfare, companion animal trade, companion animal shelters, killing of companion animals, protection of companion animals, companion animal theft*” were searched. Two hundred fifty decisions of the Court of Cassation directly related to these headings and concerning Law No. 5199 were reached. It was observed that a direct reference was made to the convention in only nine of these decisions (Figure 2). Two hundred sixteen of the decisions include the subject of animal theft. The crime of theft against animals has not been regulated in Law No. 5199 in Turkish law and has only been regulated in the TPC. Twenty five decisions are related to the killing of animals regulated in Law No. 5199. The regulation regarding the killing of animals was regulated in the TPC prior to 2021; during this period, animals were considered “property,” and after 2021, this understanding changed with the understanding that animals are also “living beings” in Law No. 5199 (17). For this reason, after reviewing the decisions made for the period following 2021, no rapid adaptation or clear reflection of those decisions was observed. The subject of the nine judicial decisions, on the other hand, is the cases regarding the keeping of animals in residences in apartments and housing estates where there is a communal life. It is observed that a direct reference is made to the convention in these nine cases in question.

The content of the nine decisions making a direct reference to the Convention among the Court of Cassation decisions where the name of the convention was searched between the years 2015 and 2025 has been evaluated in terms of having a positive or negative impact on Animal welfare. As can also be understood from the decision contents below, the Turkish Court of Cassation, in cases regarding the permission for the Keeping of animals in residences in apartments and housing estates where there is a communal life, has generally not accepted the provisions of the international convention used by the plaintiff or the defendant as a means of defense (Table 3). Six of these decisions resulted in the direction of the removal of the animals from the residences, that is, against Animal welfare. Three of them allowed the animals to live in the residences by adhering to the provisions of the international convention (*y*= *Supreme Court, hd*= *Civil Chamber, hgk*= *General Assembly of the Supreme Court*).

**Table 3:** The content of decisions referencing the convention.

Subject	Decision taken regarding animals	Merits Number of the Court of Cassation Decision
Keeping animals in the apartment	negative	y5hd 2022/15608
Removal of an animal kept in a communal living area	positive	y5hd 2025/997
Keeping three dogs in a communal living area	positive	y5hd 2025/1224
Keeping animals in a communal living area	positive	y18hd 2014/6465
Removal of a dog from a communal living area	negative	y18hd 2015/10963
Keeping a dog in an apartment	negative	y20hd 2017/836
Discomfort of the flat owners due to keeping a dog in a communal living area	negative	y20hd 2017/4850
Keeping a dog in a residence	negative	y20hd 2018/1498
Keeping a dog in a communal living area (prohibited in the condominium management plan).	negative	y hgk 2017/3018

Before examining the negative decisions of the Turkish Court of Cassation regarding *animal welfare* and their justifications, there is an issue deemed beneficial to state. In Turkish law, the *keeping of animals* in areas where there is a communal life depends on the decisions in apartment management plans. If the plan states that animals cannot be kept, and if there is an objection regarding the keeping of animals in that residence, this situation is taken to court (17, 34). As stated above, international conventions have the force of law. Therefore, written sources such as apartment management plans cannot be contrary to the laws. The evaluation of the Turkish Court of Cassation on this subject, however, is that there is no provision either in Law No. 5199 or in the international convention stating that companion animals can be kept in the independent sections of residences where there is a communal life. Therefore, due to the absence of a provision on this matter in the international convention, the Turkish Court of Cassation primarily accepts apartment management plans.

The reason for having judicial decisions on limited subjects regarding animal rights and animal welfare is that administrative fines are imposed as sanctions for the violation of the provisions related to Animal welfare, protection, and leading a healthy and sustainable life regulated in Law No. 5199. The subjects that go to the judicial justice system are very limited. For example, while the Killing of animals is the subject of the judicial justice system, performing Surgical operations on animals is subject to an administrative fine, and this sanction is applied by the competent administrations, which means that these matters are not taken to court.

**Figure 3:**
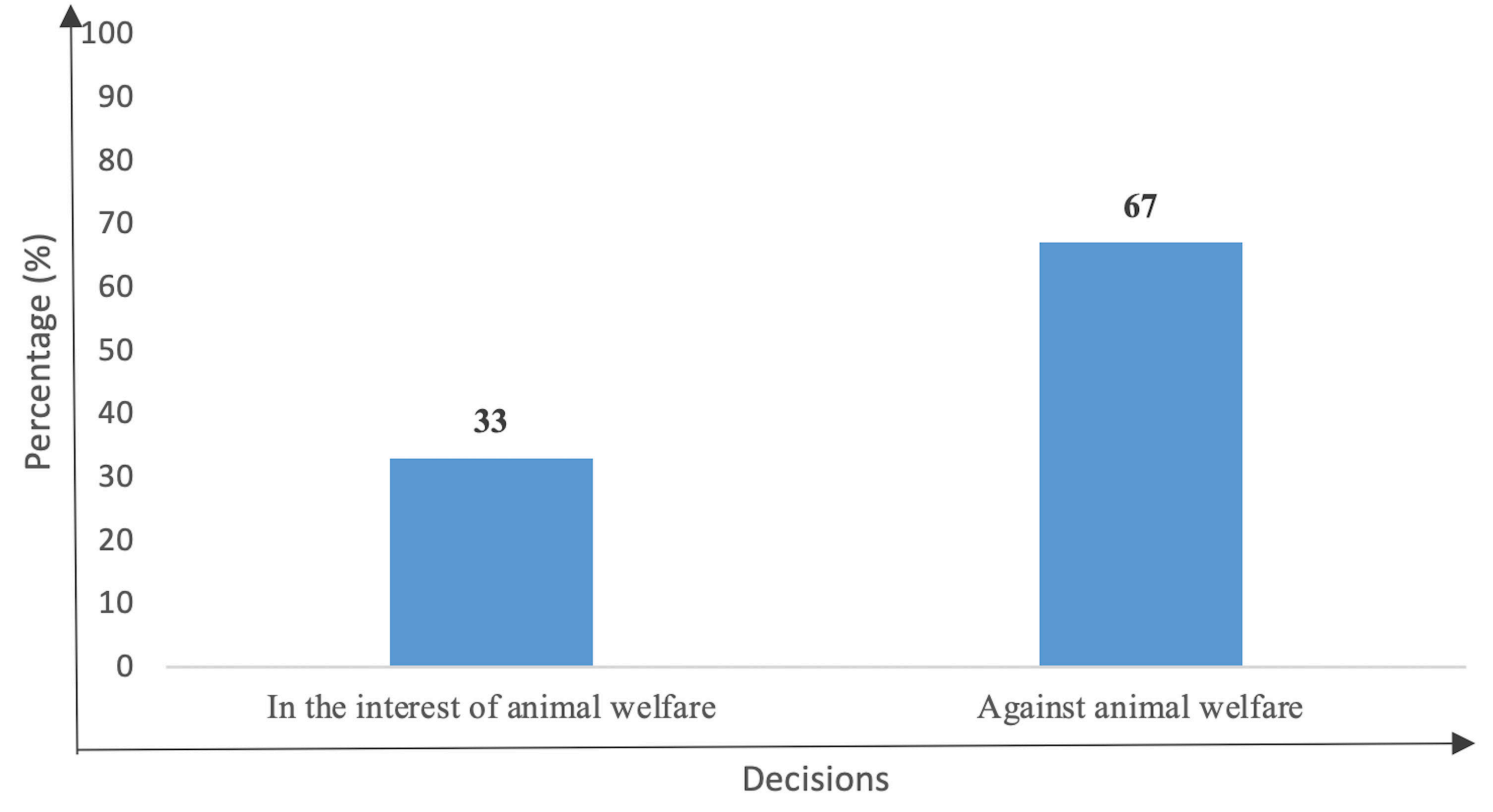
Analysis of Judicial decisions referring to the convention.

The issue of why decisions against the Convention emerge while Law No. 5199 shows a high level of compliance with the Convention at the rate of 77% is worth examining. It is observed that in disputes arising regarding animals kept in communal areas, the decision is given against the animals in the form of their removal, on the grounds that, per the provision encountered in Turkish judicial decisions, there is no provision in the international convention stating that companion animals can be kept in communal areas (Figure 3).

The reason why the court decisions encountered in the examined judicial decisions are specifically on certain subjects is due to the fact that the sanctions of Law No. 5199 are administrative sanctions, and because these sanctions are applied by the administration, the matter does not reach the judiciary for resolution.

### 3.3 SWOT analysis

The SWOT table, which was formed as a result of evaluating the analysis applied to Law No. 5199 according to the Convention together with interviews with subject-matter expert scientists, outputs of attended academic meetings, official institution data, conducted legal content analysis, mainstream media news, social responsibility project reports, etc., is as follows (Table 4).

**Table 4:** SWOT analysis of considered data.

Strengths	Availability of administrative structuring. Being a party to the Convention. Existence of laws and secondary legislation regarding Animal welfare. Presence of sanctions. High awareness within the society. Effectiveness of NGOs (non-governmental organization). Effective use of social media. Inclusion of a curriculum regarding Animal welfare in education.
Weaknesses	Absence of a provision in the law regarding the Keeping of animals in communal living areas. Lack of administrative personnel regarding the implementation of Law No. 5199. Authority confusion among competent administrations regarding Animal welfare (district municipalities, ministry, etc.). Inadequacy of Animal sanctuaries. Ineffective execution of animal sterilization policies. Lack of effective methods and tools regarding animal adoption. Irregularity in preventing the increase in the number of stray animals on the streets.
Opportunities	Social sensitivity of the society regarding animal rights. Ability to track animals through microchipping. State support for vaccination and identification. Provision of food and shelter for animals through campaigns. Public availability of provincial commission reports regarding Animal sanctuaries. High level of awareness among NGOs regarding animal rights. Social media posts forcing decision-makers to take action.
Threats	Increase in animal numbers and its damage to wildlife, especially in areas close to forests (risk of hybridization, food competition, spread of diseases, etc.). Failure in sterilization applications due to the increase in uncontrolled breeding. Negative reactions in the society toward stray animals. Negative attitude and pressure from a certain segment of society toward animal rights defenders. Dangers to humans and other living beings that may arise in the environment with the increase in animal numbers (epidemics, aggression, etc.). Spread of diseases originating from stray animals.

**Strategies in terms of strengths and opportunities:** it can be stated that the administrative structuring will attract more public power by making an efficient division of labor and implementing productive practices on the subject. Furthermore, thanks to the more effective sanctions introduced with the updates made to the law, especially in 2021, the maintained and controlled keeping of companion animals has become more regulated. With the power of NGOs and social media, food and shelter-building campaigns for stray animals can be carried out in coordination with wider audiences. Applied courses introducing companion animals with a focus on the love of nature can also be defined in the curriculum. Legal action can be taken by ensuring faster communication through the power of social media regarding animals in distress. It is known that regular training and inspections are conducted in places engaged in the animal trade, such as pet shops.

**Strategies in terms of weaknesses and opportunities:** the uncertainty regarding the conditions under which the Keeping of animals will be conducted in communal living areas, which is a matter of high social sensitivity in Türkiye, needs to be resolved by making a regulation in the relevant article of the law. Additionally, by increasing the number of personnel working in Animal sanctuaries, it should be ensured that they receive regular training on issues such as sterilization, treatment, and adoption.

**Strategies in terms of strengths and threats:** personnel working with an ecosystem-oriented approach should be present to address risks such as hybridization in wildlife, food competition, and disease due to the abandonment of companion animals in areas close to forests. Along with the increase in the number of stray animals and the increase in crimes committed against animals, announcing examples of cases that received sanctions through official social media accounts is deterrent and effective. Taking environmental precautions, such as occupational safety, in applied courses aimed at animal love or identification in the education curriculum varies depending on the awareness level of the institutions.

**Strategies in terms of weaknesses and threats:** local governments and the Ministry often draw public reaction regarding practices such as population reduction and sterilization of stray animals. Determining a clear application framework and program on this issue and announcing it to the public will ensure that the administration and the public act together in sterilization or placement in Animal sanctuaries. It is important to effectively monitor the incorrect practices of individuals engaged in commercial animal breeding, such as breeding hybrid species with wild animals. Wild animals are under state protection and legal guarantee through a separate legislation. The public's lack of information regarding Animal sanctuaries leads to confusion by shifting the responsibility solely to the administration. It is known that activities such as making animals fight under the name of entertainment and using animals in transportation in a way that exceeds their strength are not sufficiently inspected.

## 4 Discussion

In all nine decisions referring to the ECPPA, the basis cited in disputes regarding the feeding/walking of companion animals in common areas was that “there is no provision in either Law No. 5199 or the ECPPA stating that companion animals may be kept in a separate unit.” In Turkish law, disputes regarding animal rights and welfare that are brought before the courts relate solely to decisions made in the apartment management plan regarding whether pets may live in communal living areas. This is because, under Article 28 of the Condominium Law No. 634, every apartment complex is required to prepare an apartment management plan approved by its residents. If the apartment management plan contains no provisions regarding the keeping of companion animals or if such provisions are insufficient, the matter becomes subject to court decision. Whether the court rules in favor or against the owners, the decisions—such as requiring the owners to move out of their residence along with their companion animals and begin the process of finding a new place to live—directly impact the animal's welfare. Court rulings do not pertain to the animals' aggressiveness; they concern solely whether keeping pets in apartment buildings or residential complexes is permitted. Furthermore, decisions that are unfavorable to the animals are subject to the provision in Article 3/2 of the ECPPA and Article 10/2 of Turkish “Regulations on the Protection of Animals,” which states, “Nobody shall abandon a companion animal.”

Furthermore, given that bull and camel wrestling involve physical contact between animals that can result in harm to their physical integrity, such cultural activities should not be permitted by law. Table 2 outlines the criminal penalties for causing harm to animals (such as Articles 10, 24, 28) but does not define the threshold at which such harm is counted as a violation. Furthermore, although Law No. 5199 contains prohibitive provisions regarding the carriage, care, transport, and breeding of animals, legal reg-ulations must be enacted to ensure effective enforcement and to make the penalties sufficiently deterrent.

The limitations of this study include the fact that it is based on the legislation of one country, the number of court decisions citing the convention is limited, and the SWOT analysis is shaped according to the natural course of events in each country. The keywords identified in this study include the Turkish Penal Code (TCK), Law No. 5199, and the provisions in the Turkish Code of Obligations concerning animals, covering crimes, compensation, and other legal issues. Since the subject of this study pertains solely to the ECPPA, court decisions in which the convention's name appears were examined. No provisions regarding theft or other crimes involving animals were found in these decisions. Therefore, the study was limited to court decisions addressing the convention.

## 5 Conclusions

It has been observed that some provisions regarding the *Killing of animals* in Turkish legislation are not compatible with the Convention principles. In the convention, it says “all other emergencies,” but in Law No. 5199 in Turkish legislation, for one species (dogs, etc.), the possibility of killing animals only in the event that they pose a danger has been granted. The Convention has not given a broad punitive authority regarding danger. The ECPPA has not made a direct evaluation regarding human health concerning this killing. The exceptions regarding Article 13 in the convention for this article comply with Turkish legislation. However, it can be said that the interpretation in Law No. 5199 is kept broader compared to the convention. In this regard, clearer and more concise conditions should be introduced.

According to the *Killing of animals* in the convention (Article 11 and 13), it is regulated that animals can be killed under certain conditions. These conditions regulate that animals can be killed in cases where the number of animals creates a problem, where diseases cannot be controlled, and in cases of preventing the suffering of animals. In Article 12 of the convention, there are provisions regarding the killing of animals in the reduction of animal numbers. In Article 13 of Law No. 5199, the reduction of numbers by killing animals when they pose a danger to environmental and human health, and the euthanasia of animals, are regulated. The condition for euthanasia in the law is expressed as unavoidable threats directed toward human and environmental health. It has been observed that the “unavoidable threats” in Law No. 5199 should also be explicitly stated in the law.

Inadequacies regarding the deficient parts in the legislation, which emerged at a rate of 23% in convention compliance, should be resolved. Seven of the 12 principles of the convention were found compatible with Law No. 5199, while five principles were found incompatible. Looking at the incompatible principles, the lack of communication between the public and the administration regarding *Animal sanctuaries* and the uncertainty of the system come to the fore. It has been observed that there is no explicit provision in the law regarding the *Breeding of animals* and *Trading and commercial breeding*. A need for increasing legal protection has also been observed regarding the *Subjecting animals to advertising, entertainment, exhibitions and competitions*; in its current state, it leaves a door open for animals to be harmed. Although the convention does not compromise on this issue, allowing “traditional shows for folkloric purposes that do not contain violence” in Law No. 5199 paves the way for organizing competitions such as animal wrestling. This article, which leads to animals harming each other, should be removed from the law. One of the most controversial issues due to its open-ended expression is the reduction of animal numbers. In this regard, the conditions required for number reduction should be determined more comprehensively in Law No. 5199. Another principle that remains weak in the law is the *Information and education programs about animals*. Education must be provided regularly and in a planned manner to the required interest groups within the framework explained in the convention. Especially pet shops and animal markets should be encouraged for such training.

It has been observed that Law No. 5199 shows full compliance and is sufficient with the majority of the Convention principles (*Animal welfare, Keeping of animals, Breeding of animals, Conditions for acquiring a companion animal, Training of companion animals, Surgical operations on animals, Killing of animals*). It has been observed that a reference was made to the convention in only 3.6% of the judicial decisions regarding the subject. Sixty seven percent of the decisions in which the convention was referenced resulted against the animals. This is a rare situation for Turkish legislation, which integrates international conventions into its domestic law well. Looking at the negative decisions and justifications of the Turkish Court of Cassation regarding *Animal welfare* with reference to the Convention, it has been observed that there is a deficiency in both the law and the Convention regarding the *Keeping of animals* in communal living areas. It has been observed that the decisions resulting negatively are directed toward disputes in apartments and housing estates, namely in communal living areas. In Turkish law, the *Keeping of animals* in areas where there is a communal life depends on the decisions in apartment management plans. If the plan states that animals cannot be kept, and if there is an objection regarding the *Keeping of animals* in that residence, this situation is taken to court. In Turkish law, international conventions have the force of law. Therefore, written sources such as apartment management plans cannot be contrary to the laws. According to the Convention, it is stated that companion animals cannot be abandoned. The evaluation of the Turkish Court of Cassation on this subject, however, is that there is no provision either in Law No. 5199 or in the international convention stating that companion animals can be kept in the independent sections of residences where there is a communal life. Due to the absence of a provision on this matter in the international convention, the Turkish Court of Cassation primarily accepts apartment management plans. Therefore, it is necessary to add an explicit provision to the Convention on this matter. In addition, an article regarding this deficiency should be added and regulated in Law No. 5199, and objective standards should be introduced for this.

Today, the reason for having judicial decisions on limited subjects regarding animal rights and *Animal welfare* in Türkiye is that administrative fines are imposed as sanctions for the violation of the provisions related to *Animal welfare*, protection, and leading a healthy and sustainable life regulated in Law No. 5199. The subjects that go to the judicial justice system in Law No. 5199 are very limited. For example, while the *Killing of animals* is the subject of the judicial justice system, performing *Surgical operations on animals* is subject to an administrative fine and this sanction is applied by the competent administrations. This situation means that judicial subjects are not taken to court. It has been observed that a provision should be added to Law No. 5199 and additional regulations should be introduced to the secondary legislation regarding this matter. Furthermore, while making these regulations, the exceptions regarding laboratory animals must be kept at the level of current international ethical consensus.

Although Law No. 5199 exists in line with the principles of the Convention, it has been found that the jurisprudence regarding this law has not been strongly formed. Even if the legislation is strong in this regard, it is seen that regulations are needed in terms of its place in sanctions and jurisprudence. With the law finding its full place in judicial decisions, social deterrence will also be in question. It is anticipated that the difficulties in practice can be resolved over time with the strategic approach obtained as a result of the SWOT analysis. According to the results obtained from the SWOT analysis, a few points need to be strengthened. The issue of *Animal sanctuaries* is regularly included in the provincial commission reports on stray animals, and this is open to public access. The public's lack of information regarding *Animal sanctuaries* leads to confusion by shifting the responsibility solely to the administration. Additionally, according to the Convention, individuals who find stray cats and dogs are encouraged to report them to the competent authorities. Conducting awareness-raising and education studies by NGOs and the administration regarding the studies carried out on this subject and what the public can do about stray animals will ensure that the administration and the public act together in sterilization or placement in *Animal sanctuaries*. Furthermore, measures to prevent wild animals and domestic animals from coming together should be taken by the administration, and local government personnel working on this subject should be identified. The lack of trained administrative personnel regarding animal rights/welfare should also be resolved.

## Data Availability

The original contributions presented in the study are included in the article, further inquiries can be directed to the corresponding author.
